# Supplementary material for: Cryobacterium Inferilacus sp. nov., a Pshychrophilic Ureolitic Bacterium From Lake Untersee in Antarctica
Source: Microorganisms. 2025 Apr 25;13(5):990. doi: 10.3390/microorganisms13050990 (PMC12113805; doi:10.3390/microorganisms13050990)
Supplement: Supplementary file 1 [file microorganisms-13-00990-s001.zip › microorganisms-3582622-supplementary.pdf]

microorganisms

MDPI

Article

Cryobacterium Inferilacus sp.nov., a Pshychrophilic Ureolitic Bacterium From a Lake Untersee in Antarctica

Yulia Yu. Berestovskaya [1], Tatiyana P. Tourova [1], Denis S. Grouzdev [2], Natalia V. Potekhina [3], Dmitry S.Kopitsyn [4], Nikolay V. Pimenov [1] and Lina V. Vasilyeva [1]

[1] Winogradsky Institute of Microbiology, Research Center of Biotechnology, Russian Academy of Sciences, Moscow 119071, Russia; jberestovskaja@mail (J.J.B.); tptour@rambler.ru (T.P.T.); lvasilyeva@mail.ru (L.V.V.); npimenov@mail.ru (N.V.P.)

[2] SciBear OU, 10115 Tallinn, Estonia; denisgrouzdev@gmail.com (D.S.G.)

[3] Lomonosov Moscow State University, Moscow, 119991, Russia; potekhina56@mail.ru (N.V.P.)

[4] Gubkin University, 65-1, Moscow 119991, Russia; kopicin.d@inbox.ru (D.S.K.)

* Correspondence: jberestovskaja@mail.ru (J.J.B.); Tel.: +7-499-135-0341

This file includes:

Figures S1 to S4

Tables S1 and S2

 https://doi.org/10.3390/xxxxx

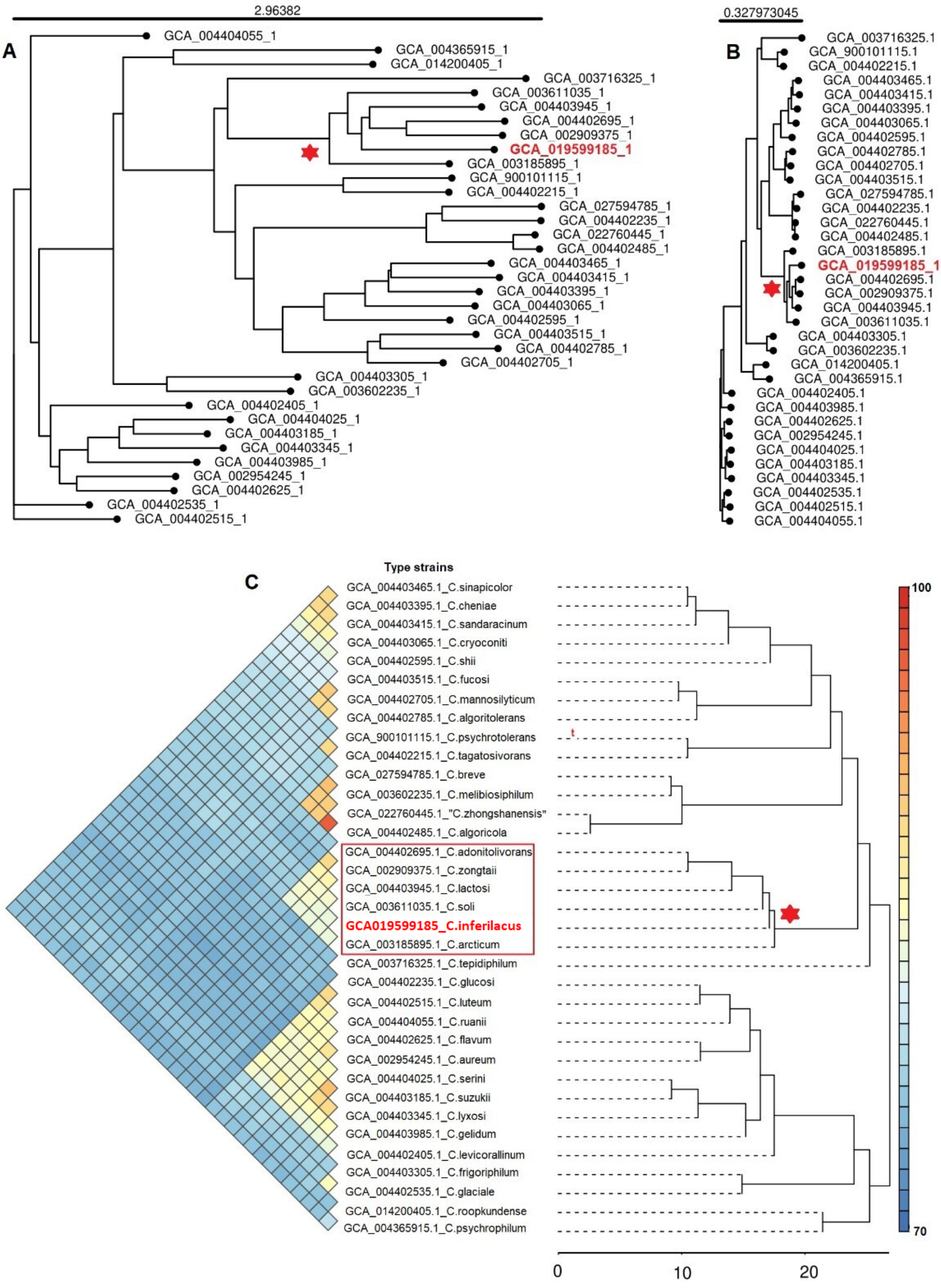

Figure S1. Pan-genome analysis of strain 1639^T and 34 other *Cryobacterium* type strains. (A) Phylogenetic tree highlighting the relationship of strain 1639^T Z129 with other *Cryobacterium* type strains based on the genome sequences. (B) Phylogenetic tree highlighting the relationship of strain 1639^T Z129 with other *Cryobacterium* type strains based on the core genes of the genome sequences. (C) Pairwise comparisons of average nucleotide identity (ANI). The color indicates the value of ANI, the value range is 90–100 with color turning from blue to red. In all trees the name of the studied strain is marked by red boldface. On all trees the clusters of species closest to this strain are marked by a red asterisks.

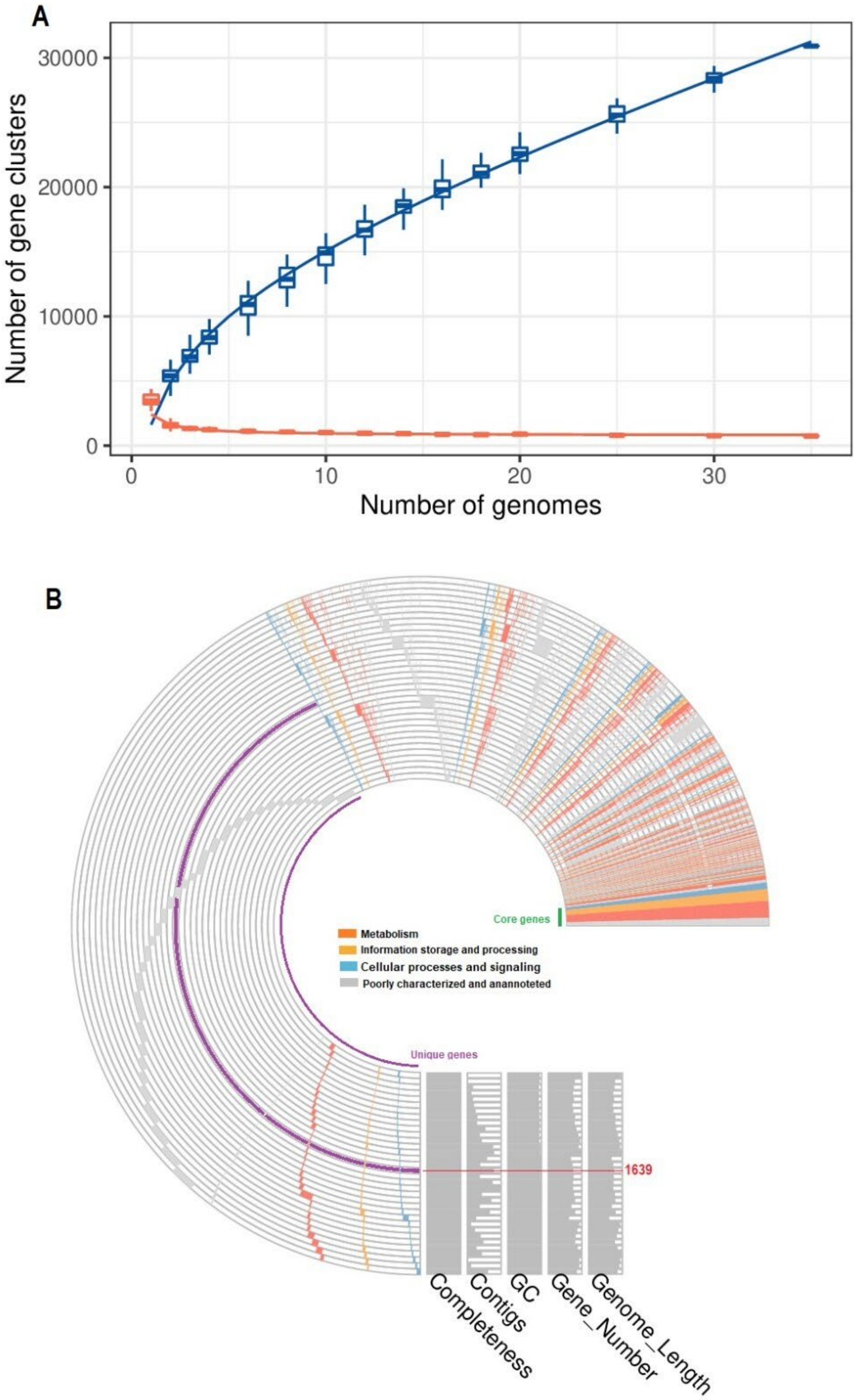

Figure S2. Pan-genome analysis of *Cryobacterium* type species calculated with IPGA. (A) The number of pan gene clusters and core gene clusters along with the addition of every new type

strains of *Cryobacterium*, including the last strain 1639ᵀ. (B) Primary COG annotation showing the core genes, unique genes, number of contigs, GC content, and genome length.

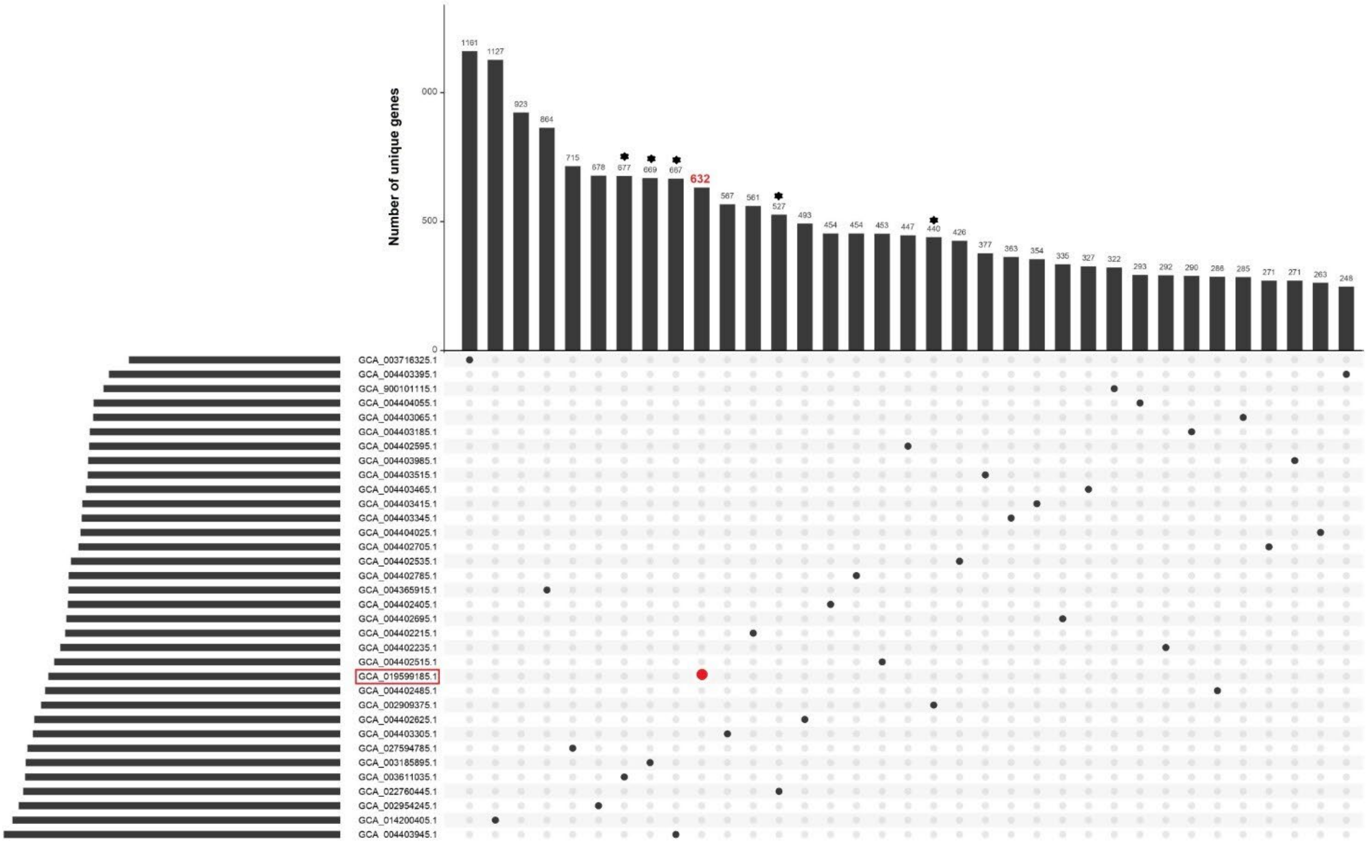

Figure S3. Distribution of unique genes among *Cryobacterium* type strains. Numbers of unique genes are marked by red boldface for the strain 1639ᵀ and by a black asterisks for the strains of the species closest to this strain.

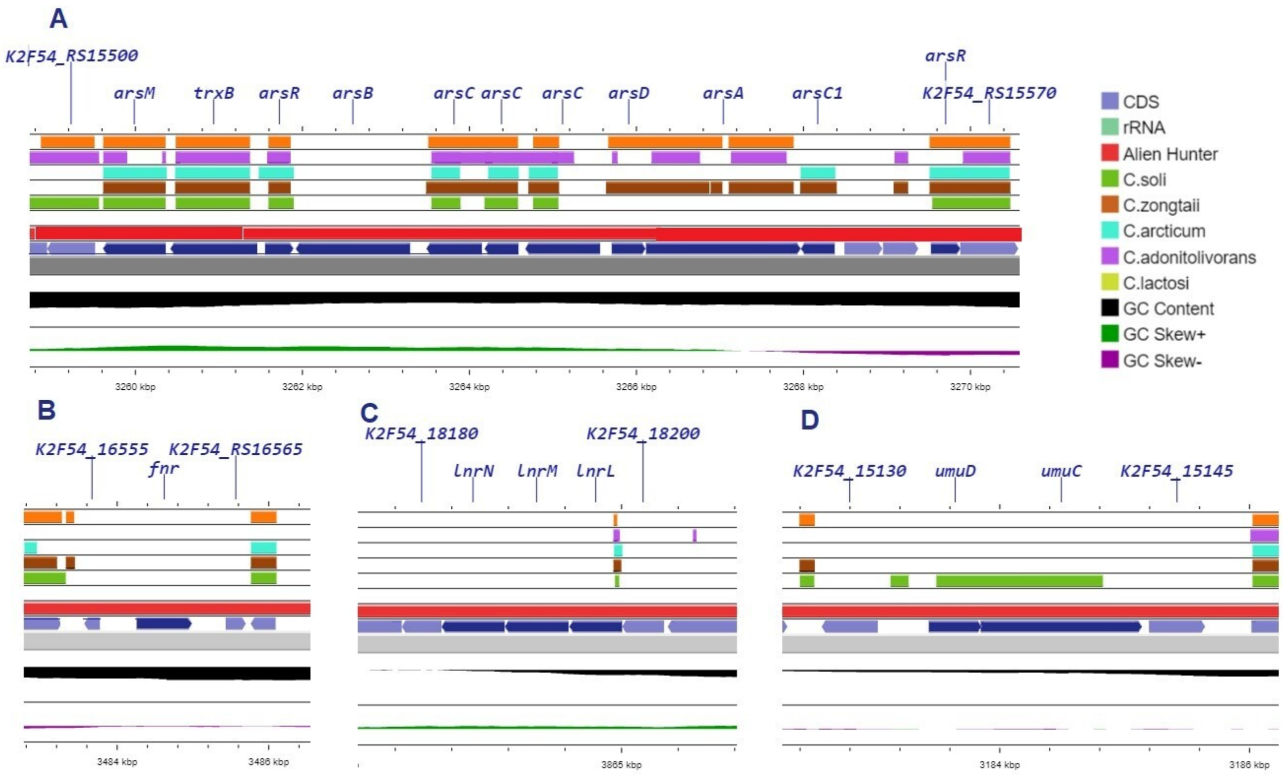

Figure S4. Localization of putative transferred genes on the circular genome map of the strain 1639^{T}. (A) the large *ars* operon; (B) gene *fnr*; (C) lnrLMN operon; (D) UmuDC operon.

Table S1. Genome parameters and relatedness indices (%) between the strain 1639T and type strains of the genus *Cryobacterium*.

Species	Type strain	Assembly Accession	GenBank Accessions	Size	GC Content	Similarity	
						dDDH	ANI
Cryobacterium inferilacus	**1639**	**GCA_019599185.1**	**JAIEUL000000000**	**4017491**	**68.0**	**100**	**100**
Cryobacterium zongtaii	**TMN-42**	**GCA_002909375.1**	**PPXD00000000**	**4048390**	**67.6**	**21,1**	**87.8**
Cryobacterium arcticum	**SK-1**	**GCA_003185895.1**	**QHLY00000000**	**4305516**	**68.0**	**40,3**	**87.0**
Cryobacterium adonitolivorans	**RHLS22-1**	**GCA_004402695.1**	**SOFL00000000**	**3669913**	**67.6**	**21,9**	**87.5**
Cryobacterium lactosi	**Sr59**	**GCA_004403945.1**	**SOHM00000000**	**4657575**	**67.6**	**33,5**	**87.7**
Cryobacterium soli	**GCJ02**	**GCA_003611035.1**	**CP030033**	**4385465**	**68.4**	**28,8**	**85.0**
Cryobacterium breve	ERMR7:08	GCA_027594785.1	CP075584	3959399	67.8	21,7	84.9
Cryobacterium psychrotolerans	CGMCC 1.5382	GCA_900101115.1	FNFU00000000	3247111	68.3	22,2	84.9
Cryobacterium roopkundense	DSM 21065	GCA_014200405.1	JACHBQ000000000	4531652	65.2	21,1	84.3
"Cryobacterium zhongshanensis"	ZS14-85	GCA_022760445.1	JALGAR000000000	4412594	67.9	28,8	84.9
Cryobacterium aureum	Hh31	GCA_002954245.1	PPTG00000000	4314199	64.3	21,9	84.3
Cryobacterium melibiosiphilum	Hh39	GCA_003602235.1	QZVS00000000	4711041	66.9	28,1	84.7
Cryobacterium tepidiphilum	NEAU-85	GCA_003716325.1	RDSR00000000	2862819	68.9	21,4	84.4
Cryobacterium psychrophilum	DSM 4854	GCA_004365915.1	SODI00000000	3677616	64.8	21,1	84.4
Cryobacterium glaciale	HLT2-23	GCA_004402535.1	SOEY00000000	3715656	65.4	20,9	84.6
Cryobacterium tagatosivorans	Sr47	GCA_004402215.1	SOEZ00000000	3783960	68.3	21,1	84.9
Cryobacterium flavum	Hh8	GCA_004402625.1	SOFD00000000	4101684	64.7	22,4	84.5
Cryobacterium levicorallinum	Hh34	GCA_004402405.1	SOFE00000000	3753316	64.5	21,1	84.4
Cryobacterium luteum	Hh15	GCA_004402515.1	SOFF00000000	3841120	65.1	20,8	84.4
Cryobacterium algoricola	MDB2-B	GCA_004402485.1	SOFG00000000	4152097	67.9	21,9	84.9
Cryobacterium mannosilyticum	RHLT2-21	GCA_004402705.1	SOFM00000000	3651597	68.6	22,4	85.0
Cryobacterium algoritolerans	MDT1-3	GCA_004402785.1	SOFP00000000	3715409	67.0	21,8	84.8
Cryobacterium glucosi	MDB1-5	GCA_004402235.1	SOFS00000000	3927216	67.7	21,8	85.0
Cryobacterium shii	TMT1-22	GCA_004402595.1	SOFY00000000	3487785	68.1	22,1	87.4
Cryobacterium cheniae	TMT2-48-2	GCA_004403395.1	SOGN00000000	3195001	67.7	22,4	85.0
Cryobacterium sandaracinum	TMT2-16	GCA_004403415.1	SOGO00000000	3510597	66.7	22,3	84.9
Cryobacterium sinapicolor	TMT1-23-1	GCA_004403465.1	SOGQ00000000	3530071	67.2	22,2	85.1
Cryobacterium lyxosi	TMT1-1	GCA_004403345.1	SOGT00000000	3539219	63.5	20,6	84.4
Cryobacterium cryoconiti	TMT1-51	GCA_004403065.1	SOHA00000000	3433919	67.8	21,9	85.0
Cryobacterium frigoriphilum	Hh14	GCA_004403305.1	SOHE00000000	4383662	66.7	21,8	84.6
Cryobacterium fucosi	Hh4	GCA_004403515.1	SOHH00000000	3438926	68.0	22,6	85.0
Cryobacterium suzukii	Sr39	GCA_004403185.1	SOHJ00000000	3459920	64.0	20,9	85.0
Cryobacterium ruanii	Sr36	GCA_004404055.1	SOHK00000000	3374308	64.4	20,8	84.4
Cryobacterium gelidum	Hz16	GCA_004403985.1	SOHL00000000	3468075	64.5	20,9	84.4
Cryobacterium serini	Sr54	GCA_004404025.1	SOHN00000000	3587208	63.8	20,5	84.3

Table S2. Heatmap of CAZy families distribution for genomes of strain 1639^{T} and other *Cryobacteriun* type strains.

Species	Type strain	GHs	GTs	CEs	AAs	CBMs	PLs
Cryobacterium inferilacus	**1639**	**50**	**54**	**8**	**8**	**3**	**0**
Cryobacterium soli	**GCJ02**	**72**	**39**	**11**	**4**	**7**	**2**
Cryobacterium zongtaii	**TMN-42**	**76**	**37**	**7**	**8**	**2**	**0**
Cryobacterium arcticum	**SK-1**	**77**	**45**	**7**	**9**	**3**	**2**
Cryobacterium adonitolivorans	**RHLS22-1**	**49**	**38**	**7**	**6**	**3**	**0**
Cryobacterium lactosi	**Sr59**	**127**	**45**	**10**	**8**	**4**	**2**
Cryobacterium breve	ERMR7:08	44	44	11	4	2	1
Cryobacterium psychrotolerans	CGMCC 1.5382	19	33	8	7	3	0
Cryobacterium roopkundense	DSM 21065	39	43	8	10	3	0
"*Cryobacterium zhongshanensi*"	ZS14-85	51	46	13	7	2	2
Cryobacterium aureum	Hh31	23	42	11	10	2	3
Cryobacterium melibiosiphilum	Hh39	49	38	5	12	2	3
Cryobacterium tepidiphilum	NEAU-85	29	29	7	3	3	1
Cryobacterium psychrophilum	DSM 4854	23	38	5	10	2	0
Cryobacterium glaciale	HLT2-23	22	37	5	10	2	2
Cryobacterium tagatosivorans	Sr47	29	37	9	10	3	0
Cryobacterium flavum	Hh8	16	47	8	6	2	2
Cryobacterium levicorallinum	Hh34	22	44	7	7	2	4
Cryobacterium luteum	Hh15	17	44	3	6	2	3
Cryobacterium algoricola	MDB2-B	45	49	13	6	2	2
Cryobacterium mannosilyticum	RHLT2-21	33	39	6	7	3	0
Cryobacterium algoritolerans	MDT1-3	32	40	5	9	2	0
Cryobacterium glucosi	MDB1-5	51	41	12	6	4	2
Cryobacterium shii	TMT1-22	27	42	6	8	2	0
Cryobacterium cheniae	TMT2-48-2	32	32	4	6	3	0
Cryobacterium sandaracinum	TMT2-16	31	38	4	2	4	0
Cryobacterium sinapicolor	TMT1-23-1	29	36	4	4	4	0
Cryobacterium lyxosi	TMT1-1	28	44	6	8	3	2
Cryobacterium cryoconiti	TMT1-51	35	40	6	8	4	0
Cryobacterium frigoriphilum	Hh14	35	42	6	11	2	1
Cryobacterium fucosi	Hh4	35	35	7	6	6	0
Cryobacterium suzukii	Sr39	21	37	8	6	2	1
Cryobacterium ruanii	Sr36	21	45	7	4	2	2
Cryobacterium gelidum	Hz16	28	43	11	6	3	0
Cryobacterium serini	Sr54	17	41	5	7	3	2
